# Looking beyond digital broadband speeds: Rural British Columbian’s experiences with internet connectivity as a basic necessity

**DOI:** 10.1371/journal.pone.0330347

**Published:** 2025-09-08

**Authors:** Kathy L. Rush, Cherisse L. Seaton, Angeliki-Iliana Louloudi, Eric P. H. Li, Khalad Hasan

**Affiliations:** 1 School of Nursing, University of British Columbia, Okanagan Campus, Kelowna, British Columbia, Canada; 2 Faculty of Management, University of British Columbia, Okanagan Campus, Kelowna, British Columbia, Canada; 3 Department of Computer Science, Mathematics, Physics and Statistics, University of British Columbia, Okanagan Campus, Kelowna, British Columbia, Canada; Adani University, INDIA

## Abstract

**Aim:**

This study examined the experience of digital connectivity among rural-living British Columbians both with and without access to high-speed Internet at home.

**Background:**

Evidence indicates that fewer rural communities have access to high-speed Internet compared to urban communities in Canada, despite government commitments to bring high-speed Internet to all British Columbians by 2027. Yet, differences within rural areas relative to those with access to high-speed compared to those with lower speeds remains a relatively unexplored area.

**Methods:**

A cross-sectional survey of rural British Columbians both with and without high-speed Internet was conducted between October 2023 and April 2024. Closed and open-ended questions gathered participants’ thoughts and experiences with digital technology access and use.

**Findings:**

Overall, 461 (M age = 56 years, 72% female) rural BC community members (47% with access to 50 + Mbps download speeds) completed the survey. Despite similar overall digital readiness, skill, and confidence using digital technology, those without high-speed Internet were older, more remote-living, reported using fewer connected devices alongside greater frustration with technology, yet had comparable frequency of Internet use except for less streaming compared to those with access to high-speed Internet. Similar themes were found among open-ended responses of both those with and without high-speed Internet access and surrounded: i) the actual and potential benefits of high-speed connectivity, and ii.) disconnects on many levels, but particularly between expectations for and reality of high-speed connectivity.

**Conclusion:**

Regardless of broadband speed, there were greater similarities than there were differences across rural community participants, with common perceptions of the benefits of connectivity amid experiences with pervasive disconnections on a number of levels.

## Introduction

Globally, access to the Internet has been acknowledged as a fundamental human right [1]. Canadians living in urban and rural communities rely heavily on wired Internet access (deemed “broadband” hereafter) and wireless mobile connectivity. It is essential for communication, economic growth, access to online services, learning and more [2,3]. As the digital ecosystem has evolved, the need for Internet connectivity has never been greater [2,4]. Yet, many rural communities still experience exclusion with respect to connectivity [5].

Internet connectivity is commonly measured according to download speed, or how fast information is delivered to the user, and upload speed, or how fast information is transmitted from the user to the Internet, measured in Megabits (or millions of bits) per second (Mbps) [6]. In 2016, Internet speeds in Canada were considered “high-speed” if they met or exceeded a benchmark of 50 megabits per second (Mbps) for downloads and 10 Mbps for uploads, commonly referred to as 50/10 Mbps. Prior to this (and since 2011) speeds of 5/1 Mbps were considered high-speed [7], highlighting the growing demand for faster broadband to match increased use. Although 95% of Canadians have access to broadband speeds of at least 50/10 Mbps, only 78% of those residing in rural communities (defined by Statistics Canada as areas with fewer than 1000 people or a population density of fewer than 400 per square kilometre) do [8]. However, in 2019, 91.7% of households in rural areas (and 99.8% of centres with populations from 1,000–29,999) had access to speeds of 5/1 Mbps [9], providing some level of connectivity, but still well below the Canadian target of 50/10 Mbps [10]. Globally, median fixed broadband download speeds averaged 101 Mbps in April 2025 [11]. Countries vary widely in average speeds, with Singapore (368 Mbps) among the highest download speeds, Syria among the lowest (3 Mbps), and Canada (244) and the United States (291) faring in between [11]. Yet, these country-level averages largely reflect urban-centric data and do not consider average speeds in rural areas. According to Ookla speedtest results, median broadband speeds were roughly three times faster among urban (252/65 Mbps) compared to rural (91/19 Mbps) Canadians in 2024 [12]

The rural-urban digital divide historically referred to uneven access to information technology (IT), including computers and access to Internet connections [13,14]. The COVID-19 pandemic and ensuing restrictions exacerbated the digital divide experienced by underserved rural communities [15]. Compounding the divide, since COVID-19 has been the digital by default strategy that many organizations and services have adopted coupled with the false assumption of universal connectivity [16]. The result has been the further exclusion of people from necessary participation in the digital world, because of an inability to interact online fully, when, where, and how they need to, or what has been called digital poverty [17]. Although academic literature has included many overlapping terms such as the digital divide, and digital equity, inclusion, and exclusion, a complex array of factors impact an individual’s ability to meaningfully interact with the digital world. While emphasis has been on infrastructure and connectivity as the foundation of digital inclusion, evidence indicates that there is more than infrastructure at play in digital poverty [18]. Beyond infrastructure (connectivity speed, Internet quality), in a comprehensive evidence-informed review, Ge et al. (2022) identified five additional key factors: socio-demographics (age, gender, education, income); place (geographic and community dimensions); skills and support (digital competence and confidence); everyday practice (frequency of Internet use, number of connected devices, connectivity interruptions, frustration); and purpose (motivation and reason for connecting; quality of access) [18].

Canada’s large land mass and dispersed rural communities has made universal broadband connectivity challenging especially in provinces with highly rural landscapes, such as British Columbia. Indeed, compared to national average, British Columbia has greater rural-urban internet discrepancy, with only 70% of rural households having high-speed access in 2023 compared to 99.6% of urban households [19]. Furthermore, recent evidence suggests that within rural areas, variations occur in broadband availability [20]. Differences within rural areas relative to broadband availability, specifically, between rural citizens above 50 Mbps and those below 50 Mbps, remains a relatively unexplored area. Unknown is how broadband high-speed access affects conditions beyond broadband that Ge et al. (2022) identified. Therefore, the two-fold purpose of this study was: i) to compare the differences between rural British Columbians with and without access to high-speed home broadband in terms of conditions beyond connectivity (socio-demographics, remoteness, skills and support, everyday practices and quality of access); and ii) to understand the connectivity experiences of rural British Columbians with and without high-speed connectivity. This study has the potential to provide governments with insights into rural issues and surface opportunities for improvement, such as unique perspectives and ways to tailor policy around local issues, rather than applying a one-size fits all solution approach.

### Research hypotheses

Hypothesis 1: Sociodemographic (e.g., age, education) differences reflecting consistent inequalities among different social groups will differ between community rural residents with and without home access to high-speed broadband target speeds.

Hypothesis 2: Community rural residents below home access to high-speed broadband targets will live in more remote rural areas than residents with access to high-speed broadband.

Hypothesis 3: Community rural residents with below home access to high-speed broadband targets will have significantly lower skills and support (readiness, confidence, competence) compared to residents with access to high-speed broadband.

Hypothesis 4: Community rural residents with below home access to high-speed broadband targets will have lower daily digital practices (frequency of Internet use, greater activity frustration/limitations, device usage – number of devices able to connect) compared to residents with access to high-speed broadband.

Hypothesis 5: Community rural residents with below home access to high-speed broadband targets will experience significantly lower trust and Internet quality compared to residents with access to high-speed broadband.

## Methods

### Design

A cross-sectional survey design was used combining both closed and open-ended questions to gain a comprehensive and in-depth understanding of the digital experiences among rural British Columbians with and without high-speed connectivity. For inclusion, our definition of rural was based on a community and hospital classification that categorized communities with limited general inpatient care and populations under 20,000 as rural [21]. Yet, we also incorporated Statistics Canada’s assessment of “remoteness” to provide within-rural variation in community populations and proximity to urban centres [22].

### Recruitment

There are over 200 rural communities in British Columbia [23], a Western Canadian Province with diverse rural geography (e.g., forests, lakes, deserts, grass plains, islands) and 75% of the region is covered by mountains [24]. Although participation was open to all rural-dwelling adults in British Columbia, several communities were targeted for advertising due to limited high-speed broadband availability, identified using the National Broadband Internet Service Availability Map [25]. The survey advertisement stated: “The Province of BC has committed to providing high-speed broadband for all British Columbians by 2027, and we want to hear from members of rural communities at different stages of the arrival of this service” and rural locales were targeted for survey advertising to encompass coastal, island, interior and northern regions to provide complete provincial geographical coverage. The study was promoted using research study platforms, through social media, both online and print community and advocacy group newsletters and newspapers, and email invitations to former rural survey participants. Print surveys were mailed both by request and using the Canada post neighborhood postal code targeting tool to mail surveys to lockboxes serving remote areas in interior and northern BC with Internet speeds < 5/1 Mbps. To encourage participation, survey participants were given a chance to enter a draw for one of three $100 and one $400 prizes. Rather than being driven by a target sample size, recruitment efforts were aimed at including rural adults with a variety of broadband speeds and geographic locations. All participants provided informed consent prior to completing the survey. Ethics approval was received from The University of British Columbia – Okanagan Behavioural Research Ethics Board (Approval #H22-01256).

### Procedures

Print survey participants submitted responses using postage paid return envelopes addressed to the university. Others completed an online survey administered using Qualtrics Survey Tool. Participants completed an eligibility question that asked “Are you 19 years old and currently (or were you in the past year) living somewhere in British Columbia that would be considered rural or remote (e.g., outside the commuting distance of a population greater than 20,000)?” and those who selected “no” exited the survey. Digital technology was defined throughout the survey as “any type of modern technology (e.g., smartphones with touchscreens, iPad/tablets, laptops, and home computers) that has the ability to connect to the Internet”.

### Sociodemographic characteristics

The survey included demographic items to measure participants age, gender, cultural background, education level and current living arrangement (i.e., living alone, with spouse/partner, with children, an adult roommate). Participants were also asked to indicate which devices they use from a list, including other (specify), and could select all that apply as well as to indicate whether they accessed the Internet via mobile (cellular) data. Several standardized and researcher-generated self-report measures were also included (described below).

### Place: provincial region and community remoteness

Participants selected their home community/location within one of the five provincial health regions. Each community or remote location was assigned a score corresponding to rurality based on Statistics Canada’s “remoteness index” (RI) [22]. The RI measures relative remoteness of Canadian communities based on population size and proximity to surrounding population centres. The RI value is a continuous score ranging from 0 to 1, where “0” represents the most accessible areas and “1” represents the most remote areas. Community RI score were classified into one of 5 categories: easily accessible (<0.1500), accessible (0.1500 to 0.2888), less accessible (0.2889 to 0.3898), remote (0.3899 to 0.5532), or very remote (>0.5532) [26].

### Skills: technology readiness, device proficiency, and technology self-efficacy

The Technological Readiness Index (TRI 2.0) [27], a 10-item Likert scale (e.g., “*I keep up with the latest technological developments in my areas of interest*”), was used to assess participant’s propensity to use and adopt new technology at home and at work. The TRI 2.0 measures both motivators (optimism & innovativeness) and inhibitors (discomfort & insecurity) of readiness using a 5-point Likert rating scale (1 = *strongly disagree;* 5 = *strongly agree*). Total TRI 2.0 scores are calculated through the reversal of the insecurity and discomfort dimensions and the average of the sums of the dimensions. Higher scores indicate higher technological readiness. The 16-item Mobile Device Proficiency Questionnaire (MDPQ-16) was used to assess device use proficiency [28]. Three items (e.g., “*Using a digital technology device I can* f*ind information about my hobbies and interests on the web”*) measured proficiency on a 5-point Likert type scale (1=*never tried;* 5=*very easily*). Finally, the Technology Self-Efficacy Scale was used to assess participants perceptions of their ability and confidence to use a new digital technology for certain aspects of their daily lives [29]. Six of 10 scale items (e.g., *I could use a new technology even if there was no one around to tell me what to do as I go”)* were included and measured on a 10-point Likert type (1 = *not confident;* 10 = *completely confident*)*.* The scale possesses high internal consistency (Cronbach’s α = .94) and construct validity [29].

### Daily practices: frequency of Internet use, number of connected devices, and connectivity limits to activity

The survey included items from Valentin-Sivico et al. (2023) and topics from Bakker et al. (2011) to create a measure of different activities people may do online. Participants were asked to rate on an 8-point Likert scale how often they or their family engage in twelve different activities (1 = *never*; 8 = *once a day or more*). In addition, a single-item measure of frustration (1 = rarely or none of the time; 4 = all the time) with Internet-connected technology in the past week (Tawfik et al., 2021) was included, along with researcher-generated single items about the number of devices participants are capable of connecting at home (0–5+), and the extent to which their online activity is limited by Internet connection speed (1 = *not at all;*10 = *completely limited*).

### Purpose: trust and quality

Two items (i.e., *I expect Internet websites/mobile applications will not take advantage of me; I believe most Internet websites/mobile applications are trustworthy*) were adapted from Corritore et al.’s (2005) online trust instrument to assess participants trust towards digital devices applications and Internet websites. Participants were asked to rate the degree to which they agreed with the aforementioned statements on a Likert-type scale that ranged from *strongly disagree (1)* to *strongly agree (7)*. The mean of the two items was computed (Cronbach’s Alpha = .66). In addition, participants were asked to “*please rate your Internet quality in your daily life*” on a scale from *poor/very inadequate* (1) to *excellent* (4).

### Digital infrastructure: internet speed

Internet speed was measured through OpenSpeedTest^TM^ (2022) or participant self-report. OpenSpeedTest^TM^ is a free-use, open-source speed test, used globally, considered reliable, accommodating of various web-browsers within different operating systems and that allowed for its integration within the survey [30]. Participants could select “*I do not have home Internet*” and were asked to “*enter any additional information to help us better understand these estimates or if left blank (e.g., because speed is unknown)*”. After completing the speed test questions, participants were asked: “*Do you currently have access to Internet at home that meets the current Canadian definition of high-speed - i.e., which is 50 megabits per second (Mbps) of download and 10 Mbps of upload (or 50/10 Mbps)*” (response options: yes, no, don’t know, and other, please explain).

### Open-ended questions

Participants were invited to respond to several open-ended questions in the survey including: “*How has your digital device use changed over time (e.g., increased and/or decreased, changes in attitude towards digital devices, etc.)? Please elaborate on what factors may have contributed to the change(s) if possible*”; *“Can you please provide an example of how a new technology has impacted your community (e.g., Online grocery ordering or virtual healthcare access during the pandemic)?”;* and “*Is there anything else you’d like to share about Internet, technology, and/or digital device use in your personal life or community?”.*

### Statistical analysis

Descriptive statistics were generated in SPSS version 29.0. Participants were categorized as having access (yes/no) to download speeds of 50 + Mbps through a combination of examining responses to the self-reported high-speed broadband question, speed test results, and open-ended speed test explanations. Chi-square tests were used to compare those with and without access to 50 Mbps broadband speeds on categorical variables, and between-subjects t-tests on continuous outcome variables. All continuous variables were deemed acceptably normally distributed, given the large sample size. Mann-Whitney U tests were used to compare those with and without access to 50 Mbps broadband speeds on frequency of going online for different activities. Two-sided *p* values are reported.

Richard and Morse’s (2012) iterative inductive thematic analysis process was used to analyze open-ended survey responses. Open coding was performed for units of meaning (e.g., words, phrases, or paragraphs), and common meaning units clustered into categories to construct an initial coding schema (KLR). A second author (AL) re-coded all open-ended responses for common themes and cross-checked the commonalities with the first coder. Any conflicts were resolved through discussion with the initial coding schema remaining consistent between coders. Open-ended responses were initially examined for those with and without 50 Mbps separately with text data extracted from survey responses entered into an Excel spreadsheet that was organized according to broadband access group. Since there were more similarities than differences between those with and without high-speed Internet, the cross-group categories were merged, and categories abstracted to construct themes to reflect both groups.

### Data cleaning

Of 629 responses, 461 were retained, as 87 were deemed to be bots (determined through a multi-step process considering of a number of factors, such as IP address location, response to an attention check question, repetitive or non-sense open-ended responses, etc.), 23 were ineligible (urban/easily accessible area), 42 were incomplete (did not progress beyond demographics), and 16 were duplicate surveys (determined through duplicate IP addresses and a comparison of demographics and responses) from the same participants. Missing data were retained as missing.

### Response rate

Response rate varied by recruitment strategy and included 23% (14/60) for mailed print surveys, 48% (129/267) response rate from former survey participants invited via email, 0.6% (9/1577 presentations) response to a paid ($25 CAN) 6-day Facebook advertisement and 6 responses to an invitation in one island community (population 489) newsletter as well as 6 responses to a 1-week paid ($11 CAN) advertisement in a print newspaper that distributes over 7800 copies to a number of rural communities across interior BC. We were unable to track the response rate of the remaining recruitment strategies (e.g., research study platforms, invitations shared through rural group newsletters and shared on 123 community Facebook pages), as these were conducted simultaneously.

## Findings

### Participant characteristics

The data were collected from 461 participants (332 women, 113 men, 3 nonbinary, 2 transgender, 1 agender, 7 prefer not to answer) who completed the survey between October 23, 2023, and April 10, 2024 (14 paper, 447 online surveys). Two participants submitted surveys along with detailed letters about their context and experiences. See Table 1 for a summary of participant characteristics. Participants’ age ranged between 22–83 (*M*: 55.79, *SD*: 14.23) with the majority identifying as European (76.1%), well educated, with post-secondary degree (86%), lived with a spouse/partner (71.8%). Despite provincial survey distribution to rural communities, most respondents reported being from the Interior/Kootenay region (49.7%). The participants came from 106 communities, ranging in population from 75 to 20,000, with a broad range of remoteness (See Table 1).

**Table 1:** Participant characteristics according to access to 50 mbps broadband download speed.

Participant characteristics	All participants (*n *= 461)	Has access to home download speeds of at least 50 Mbps (*n* = 216)	Does not have home download speed of 50 Mbps (*n* = 193)	Chi-Square	*df*	*p*
**Age** *M* (*SD*)	55.79 (14.2)	52.67 (14.8)	59.49 (13.5)	–	–	–
**Age groups** *n* (%)				22.96	4	<.001
20 - 34	37 (8.0)	26 (12.1)	8 (4.2)			
35–49	121 (26.2)	68 (31.6)	38 (19.9)			
50–64	157 (34.1)	70 (32.6)	66 (34.6)			
65–79	133 (28.9)	48 (22.3)	73 (38.2)			
80+	9 (2.0)	3 (1.4)	6 (3.1)			
Missing	4 (0.9)	–	–			
**Gender** *n* (%)				2.63	1	.105
Woman	335 (72.7)	161 (74.5)	131 (67.9)			
Man	113 (24.5)	50 (23.1)	55 (28.5)			
Other (e.g., nonbinary, transgender)	6 (1.3)	2 (1.0)	4 (2.0)			
Missing (includes prefer not to answer)	7 (1.5)	3 (1.4)	3 (1.6)			
**Cultural background** *n* (%)				1.35	1	.245
[Western] European descent	351 (76.1)	160 (74.1)	152 (78.8)			
Indigenous, First Nation or Metis	19 (4.1)	8 (3.7)	7 (3.6)			
Indigenous and European	10 (2.2)	7 (3.2)	2 (1.0)			
East Asian	5 (1.1)	3 (1.4)	1 (.5)			
Mixed cultural background	4 (.9)	3 (1.4)	1 (.5)			
Middle Eastern	3 (.7)	1 (.5)	2 (1.0)			
South Asian	2 (.4)	1 (.5)	1 (.5)			
East Asian and European	2 (.4)	1 (.5)	1 (.5)			
Other: Canadian	33 (7.2)	16 (7.4)	14 (7.3)			
Other (e.g., North American, USA)	7 (1.5)	3 (1.4)	4 (2.1)			
Missing (includes prefer not to answer)	25 (5.5)	–	–			
**Education** *n* (%)				3.13	5	.680
** **Some high school or less	10 (2.2)	3 (1.4)	5 (2.6)			
** **Completed high school	42 (9.1)	24 (11.1)	14 (7.3)			
** **Trades or college diploma	110 (23.9)	54 (25.0)	47 (23.4)			
Some college or diploma	93 (20.2)	41 (19.0)	39 (20.2)			
University	113 (24.5)	53 (24.5)	44 (22.8)			
Professional or graduate degree	85 (18.4)	38 (17.6)	40 (20.7)			
Missing (includes prefer not to answer)	8 (1.4)	–	–	–	–	–
**Living arrangement** *n* (%)						
Alone	88 (19.1)	39 (18.1)	38 (19.7)	0.18	1	.673
Spouse or partner	335 (72.7)	164 (75.9)	138 (71.5)	1.03	1	.310
Adult roommate	20 (4.3)	7 (3.2)	10 (5.2)	0.96	1	.326
Parents	17 (3.7)	7 (3.2)	5 (2.6)	0.15	1	.697
Child(ren) or youth	84 (18.2)	46 (21.3)	31 (16.1)	1.83	1	.176
Other (e.g., living on the same land)	3 (.7)	0 (0)	3 (1.6)	–	–	–
Missing (includes prefer not to say)	2 (.4)	–	–	–	–	–
**Devices** *n* (%)						
** **Desktop or Laptop	427 (92.6%)	197 (91.2%)	181 (93.8%)	0.97	1	.325
Smartphone	429 (93.1%)	205 (94.9%)	174 (90.2%)	3.39	1	.066
Tablet	289 (62.7%)	129 (59.7%)	125 (64.8%)	1.10	1	.294
Smart watch	144 (31.2%)	71 (32.9%)	57 (29.5%)	0.53	1	.468
Other (e.g., gaming, smart TV/speaker)	37 (8.0%)	17 (7.9%)	20 (10.4%)	0.77	1	.380
**Cellular/mobile data** *n* (%)	284 (61.6%)	149 (68.9%)	127 (65.8%)	0.47	1	.493
**Area of BC** *n* (%)				11.93	3	.008
Interior region	229 (49.7)	109 (50.5)	88 (45.6)			
Northern region	63 (13.7)	24 (11.1)	32 (16.6)			
Island region	116 (25.2)	47 (21.8)	58 (30.1)			
Coastal/Fraser Valley region	53 (11.5)	36 (16.7)	15 (7.8)			
**RI Classification** *n* (%)				8.18	3	.042
Accessible area	44 (9.5)	29 (13.4)	10 (5.2)			
Less accessible area	128 (27.8)	59 (27.3)	56 (29.0)			
Remote area	221 (47.9)	98 (45.4)	95 (49.2)			
Very remote area	68 (14.8)	30 (13.9)	32 (16.6)			

Notes: Pearson Chi-Square p-value is reported. For gender, comparison is for men and women. For cultural background, comparison is between European descent and all others. RI: Remoteness.

### Internet speed

**Fig 1:**
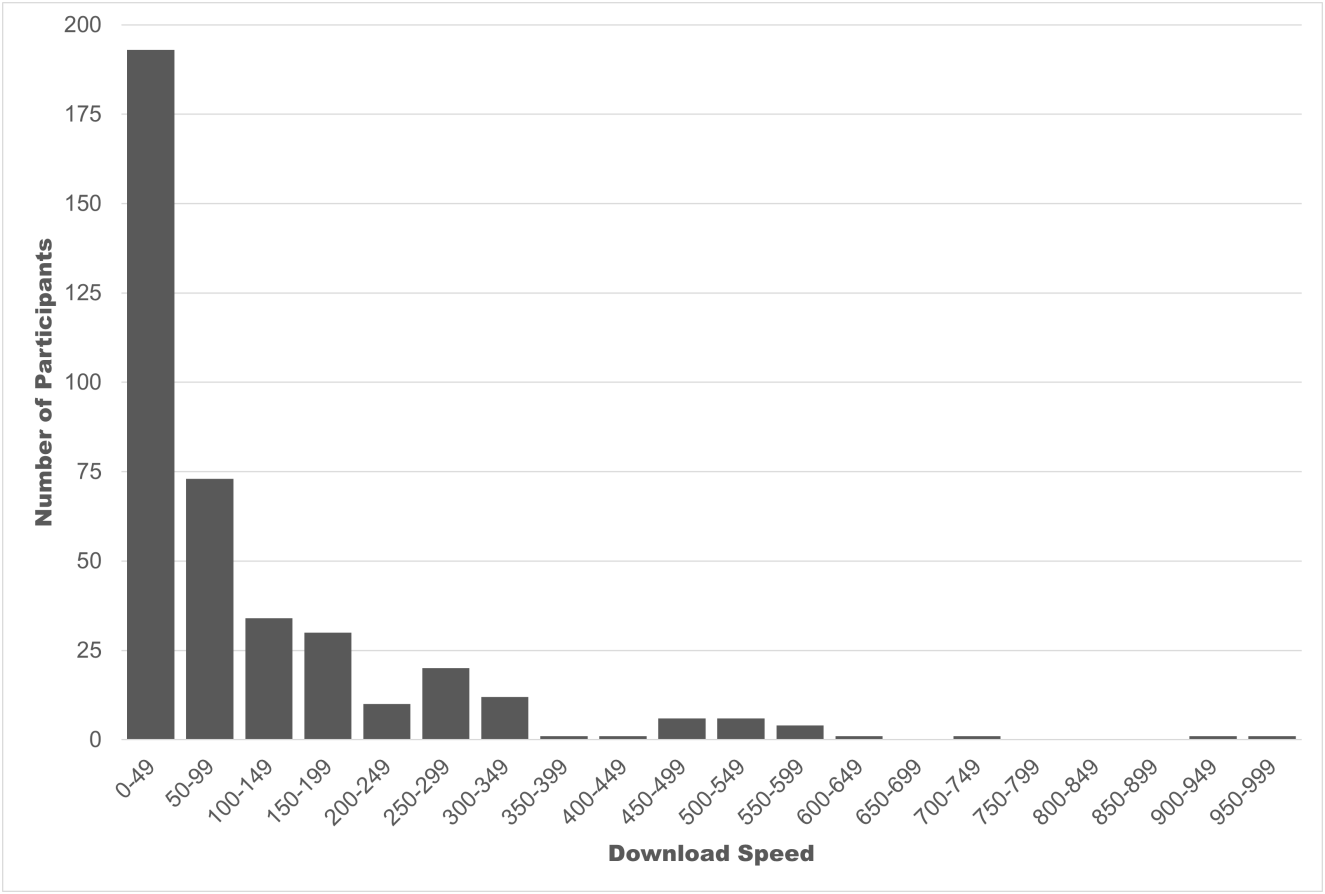
Frequency of participant download speeds.

Overall, 216 had access to home broadband download speeds of over 50 Mbps, whereas 193 did not, and 9 did not know (43 missing speed data). There was great variation in access, with several (n = 8) reporting no home Internet, and relying on work or public Wi-Fi or mobile data. Of those with access to under 50 download speeds, there was good representation among lower access speeds, though most (n = 70) had download speeds in the 25–49 Mbps range, followed by 59 with 10–24.9 Mbps, 27 with 5–9.9 Mbps, 10 with 1.5–4.9 Mbps, 8 had under 1.5 Mbps, and 11 participants had under 50 Mbps, but actual speed was unknown. Those with access to over 50 Mbps also varied greatly, with download speeds ranging from 50 to 959 Mbps. See Fig 1 for a frequency histogram displaying participant download speeds. Even among those with over 50 Mbps, speeds clustered closer to the lower end, with the majority (n = 73) reporting download speeds between 50 and 99. Average download speed did not differ significantly by region (p = .110), with Coastal/Fraser highest (Mean download speed = 154 Mbps) followed by Interior (Mean = 109 Mbps), Island (Mean = 108 Mbps), and Northern with the lowest average speed (Mean = 82 Mbps).

### Comparing those with and without high-speed internet

#### Hypothesis 1: between group differences in sociodemographic variables [age, gender, education].

Overall, those with 50 + Mbps and those below 50 Mbps were comparable on sociodemographic variables (See Table 1). Only age was lower, on average, among those with 50 + Mbps compared to those with lower speeds.

#### Hypothesis 2: between group differences in place [remoteness index, region].

There was a significant between-group difference in the remoteness index, with more of those in less accessible and more remote regions with below 50 Mbps. High-speed access also varied by region, with fewer in Northern and Island regions having access to 50 Mbps compared to Fraser Valley and Interior regions (See Table 1).

#### Hypothesis 3: between group differences in skill [digital readiness, mobile device proficiency, and self-efficacy].

There were no significant between group differences (See Table 2), in digital readiness, mobile device proficiency, and self-efficacy among those with below 50 Mbps compared to those with 50 + Mbps.

**Table 2:** Differences in continuous study variables according to access to 50 Mbps broadband download speed.

Continuous variables (response range)	All participants (*n *= 461) *M* (*SD*)	Has access to home download speeds of at least 50 Mbps (*n* = 216) *M* (*SD*)	Does not have home download speed of 50 Mbps (*n* = 193) *M* (*SD*)	Cohen’s d [95% CI]	*t*	*df*	*p*
TRI-2.0 (1–5)	3.07 (0.66)	3.11 (0.68)	3.03 (0.64)	.131 [−.06,.33]	1.33	407	.186
MDPQ – mean use score (1–5)	4.54 (0.57)	4.57 (0.50)	4.51 (0.65)	.095 [−.10,.39]	0.962	407	.337
Technology self-efficacy (1–10)	7.44 (1.79)	7.61 (1.71)	7.34 (1.83)	.152 [−.05,.35]	1.51	392	.132
Frequency of going online for:							
Social media/networking	7.40 (1.52)	7.42 (1.43)	7.39 (1.61)	–	–	–	.701
Stream videos (e.g., TV, movies)	6.44 (2.30)	6.71 (2.09)	6.16 (2.48)	–	–	–	.032
Local community information	6.21 (2.27)	6.05 (2.37)	6.39 (2.13)	–	–	–	.218
Work from home	4.93 (3.10)	5.00 (3.10)	4.86 (3.10)	–	–	–	.774
Search for health-related information	4.77 (1.64)	4.71 (1.65)	4.83 (1.61)	–	–	–	.427
Search for education information	4.48 (2.46)	4.44 (2.51)	4.55 (2.41)	–	–	–	.715
Video call/conferencing	4.37 (2.31)	4.46 (2.30)	4.27 (2.31)	–	–	–	.432
Access government services	4.01 (1.43)	4.12 (1.48)	3.89 (1.35)	–	–	–	.212
Distance learning	3.32 (2.36)	3.30 (2.36)	3.33 (2.35)	–	–	–	.878
Access emergency preparedness	3.06 (1.39)	2.95 (1.27)	3.17 (1.49)	–	–	–	.148
Use online patient portal	2.97(1.58)	2.95 (1.53)	2.98 (1.64)	–	–	–	.953
Search/apply for jobs	2.39 (2.01)	2.49 (1.95)	2.31 (2.09)	–	–	–	.050
Frustration with technology (1–4)	2.04 (0.95)	1.81 (0.89)	2.28 (0.97)	−.510 [−.71, −.31]	−5.08	397	<.001
Number of connected devices (0–5)	3.61 (1.38)	4.09 (1.19)	3.11 (1.36)	.771 [.54, 1.00]	6.72	303	<.001
Limited in online activity (1–10)	4.09 (2.92)	3.16 (2.61)	5.16 (2.89)	−.727 [−.73, −.93]	−7.25	383	<.001
Trust (0–7)	3.24 (1.35)	3.24 (1.41)	3.26 (1.24)	−.016 [−.21,.18]	−0.16	407	.876
Internet quality (1–4)	2.89 (0.72)	3.10 (0.65)	2.63 (0.71)	.703 [.50,.91]	6.93	374	<.001

Notes: TRI = Technology Readiness Index; MDPQ = Mobile Device Proficiency; frequency of going online for different activities response choices included 1 (never), 2 (once a year), 3 (several times a year), 4 (once a month), 5 (several times a month), 6 (once a week), 7 (several times a week), 8 (once a day or more); although means are reported, p values are reported from Mann-Whitney U tests for the ordinal frequency of going online variables.

#### Hypothesis 4: between group differences in daily practices [frequency of Internet use, frustration with technology, number of connected devices, online activity limitations].

Also in Table 2 it can be observed that overall frequency of Internet use did not differ between the two groups except for a lower frequency of streaming among those with below 50 Mbps compared to those with 50 + Mbps. Those below 50 Mbps download speeds at home had significantly higher frustration with technology, reported having fewer connected devices, and were more limited in online activity compared to those with access to 50 + Mbps.

#### Hypothesis 5: between group differences in purpose [Internet quality].

Although trust did not differ, there was a significant difference in Internet quality between those with 50 + Mbps compared to those below 50 Mbps with the former experiencing greater Internet quality (See Table 2).

Overall, more similarities than differences characterized participants with and without access to high-speed Internet, with no between group gender differences (See Table 2), digital readiness, mobile device proficiency, self-efficacy, and frequency of Internet use (with the exception of frequency of streaming). However, those below 50 Mbps download speeds at home had higher frustration with technology, reported having fewer connected devices, were more limited in online activity and reported lower Internet quality compared to those with access to 50 + Mbps.

### Open-ended responses

Of the 461 participants, 353 (76.6%) provided responses to open-ended questions. Open-ended responses reflected that access to high-speed Internet was not clear-cut. Similar to the quantitative analysis, there were greater similarities than there were differences across participants with and without access to 50 + Mbps home broadband. Themes highlighted participants accounts of actual and potential advantages of high-speed connectivity to benefit their community, and their experiences of pervasive disconnections on a number of levels and navigating these.

### Advantages of high-speed connectivity: actual and potential

Participants prefaced their descriptions of the perceived advantages of high-speed with the *necessity* of the Internet to everyday life, especially for rural and isolated locales where it provided the only connection to the outside world. According to one participant, “*The Internet has become as much of a utility as telephone, electricity, radio and television, especially here*” (P1018, no access to home Internet). Participants lauded actual benefits of the Internet and connectivity for individuals, families, and communities alike. Most importantly, it allowed communication and staying connected to the community through online community groups (special interest groups such as bear sightings), websites and social media to stay informed about events, programs, resources, and support needs within the community, and gaining access to news and weather and road condition reports. The benefit of staying connected outside the community especially for those living very remotely was also raised, “*In [name removed] community, we all live off the grid so having the Internet and Internet-connected phones really connects us with each other and with the outer world*” [P1069, recent access to 50 + Mbps]. Specific to the family, one parent talked about the educational value of connectivity, “*I think it is wonderful. Both my children watch kids YouTube and have learnt so much from it. I continue to learn new apps and ways to find information*” [P245, 127/10 Mbps]. Individual participants highlighted personal benefits of staying connected with family and friends, ordering online books from the library, and entertainment.

It was common for participants to reference benefits of connectivity applied to specific areas of life such as health and education. Participants singled out connectivity’s benefit in meeting health-related needs at a distance that ranged from general mental and physical well-being, to collecting biometric data for chronic disease management using digital devices to share with a specialist. For example, a participant with diabetes described the benefit of connectivity for their health care, “*Digital device iPhone has enabled me to use a CGM during medication adjustment for diabetes…and have real-time data to discuss at a Zoom a lot with my endocrinologist*” [P110, 11/0.3Mbps].

Rural participants projected the great potential for high-speed connectivity to advance their community’s economy, catalyzing job creation, and facilitating affordable rural housing opportunities. They viewed greater potential for optimizing connectivity to improve education quality in their communities. One participant spoke about enhancing bandwidth to allow multiple device use in their rural schools [P528, 54/3 Mbps] while another, an educator, described student success and children reaching their potential through inclusivity with better Internet and tech/tools [P451, 56/62 Mbps]. Another participant elaborated on the potential to leverage better streamlined and coordinated healthcare services through electronic tools such as patients’ personal health records to enhance their control of their healthcare and the use of advanced technologies such as AI that could streamline and coordinate services that would improve health care [P437, 943/937 Mbps].

### Pervasive disconnection

At the same time as participants extolled the benefits, they countered with their experiences of pervasive disconnection. The disconnection occurred at three levels: i) total/intermittent disconnection from high-speed service, ii) disconnection between Internet connectivity and daily roles and activities, and; iii) disconnection between expectations and reality.

#### Total/intermittent disconnection from high-speed service.

The erratic connectivity was compounded by frequent power outages with some communities “*going out often for 6-8 hours at a time*” [P246, 11/2 Mbps), or experiencing “*multiple long power outages each year*” [P413, 90/7 Mbps]. Topographical (e.g., trees, mountains) and seasonal factors disrupted connectivity. Even participants with over 50 Mbps relayed that during peak community use times and load on the system, connectivity was drastically reduced. For example, one participant with 65/20 Mbps considered the speed to be “*pathetic, when it even works*” and goes on to say *“[Our] Internet is old copper system with no hope of ever upgrade. Stuck in a time warp in [community name removed] with no chance to ever get fiber here*” (P514).

For several participants with access to home broadband, the lack of good cell service connection and coverage was frequently highlighted as a major disruption and inconvenience. They described “*much of this area has no cell service*” [P136, 62/8 Mbps], or “*[there are] a lot of dead zones on the highways around here. You can drive for 1 hour and no service*.” [P262, 113/3 Mbps], or “*Cellular phone signals are blocked by a nearby geographic feature*.” [P408, 277/43 Mbps]. For many this meant access/coverage only when on their properties and/or specific locations on their property (e.g., porch) to get cell service or having to drive to get cellphone connections.

#### Disconnection between access to connectivity and daily activities/roles.

High-speed disconnection, whether complete or unreliable, and daily role and activities was a recurring theme for participants. For example, participants described disconnection between having access to greater connectivity but being more disconnected from in-person communication and engagement contributing to alienation, mental health impacts, and reducing children’s outdoor community activity. One participant described the simultaneous tension between the advantages of connection but its contribution to disconnection, *“Finding information is quicker and easier but I feel it has disconnected people and ruined the way we communicate. I’d bet it contributes a lot to the state of our mental health problems. And so much more, I could go on and on*” [P152, 47/49 Mbps].

Working from home was another activity that was challenging owing to the disconnect between unstable connectivity and the speed that was needed. Participants working from home and those seeking potential work from home job opportunities described this disconnection. Those working from home found it “*really difficult*” [P298, 85/18 Mbps] and in some cases “*had to leave the community to get to an office that had power and Internet to work properly*” [P413, 90/7 Mbps]. Unreliable Internet left others unable to pursue employment from home and either continue to travel long distances to work, or relocate as one participant shared, “*My son moved away because he can’t work here due to poor Internet speeds*” [P541, 11/0.7 Mbps].

Participants expressed considerable concern about the disconnection between Internet access and carrying out other daily activities such as travel between communities and physical activity because of safety risks. They highlighted the inability to access emergency services when they were off their properties, or travelling between communities due to spotty service, and when there were power outages so “*if anyone needs help no one will know but you!*” [P1046, 179/81 Mbps] One participant even talked about no longer distance running as “*no ability to obtain help thru cell*” [P569, 81/82 Mbps]. Others described their community as “*falling behind day by day*” (P268, 15/2 Mbps) as a consequence of poor Internet access, with the inability to stream videos being commonly mentioned.

#### Disconnection between expectations and reality.

Partly driven by the pandemic, participants spoke repeatedly about the growing expectations from all sectors (government, businesses, healthcare) for citizens to be well connected and know where and how to access goods, services/programs, and resources online and yet for many in their communities this was not a reality. There were strong emotional reactions to the disconnection between expectations and reality. According to one participant, “*I am highly offended by BC Services security requirements which are beyond the scope of many people to access their own information*” [P88, 6/0.7 Mbps] and for another, “*I resent that a smartphone is required to get grocery discounts and retail info. Smartphones are expensive, fragile and the destroyer of interpersonal relationships*” [P301, 42/15 Mbps]. A participant from a very remote location described the growing societal expectation to be connected from multiple service providers who went ‘paperless’ expecting him to download bills from the Internet, which he had no access to, even though they “*never inquired whether or not I had Internet service, would never answer the phone when I called them to tell them to mail me my phone bill, disconnected my phone for nonpayment and took me several months to straighten out the mess that they solely created*” [P1018, no access to home Internet].

One participant highlighted the frustration, the point of overconsumption, and the time consumption faced when dealing with technologies:

“*The increase in the incorporation of cell phones as a requirement to interact with banks, cars, and other daily needs that never needed them before is quickly making life much more complicated than it needs to be. I find that people are spending more time using this technology OR trying to figure out how to use or fix it, than actually taking the time to enjoy the moment. There really is no need for society to go this route other than for tech giants to push their tech into new areas to make money*” [P103, 198/59 Mbps].

Participants tended to target specific vulnerable groups (e.g., older adults, houseless citizens) who they regarded as having an accentuated disconnection between expectation and reality for a variety of reasons, including lack of equipment (e.g., cell phones, laptops), digital literacy and training/support, affordability and age-related changes for interacting with technology. Older adults were often singled out because of their large proportionate representation in rural communities and the multiple barriers they face. One participant described the disconnect between online resources and age-related needs of older adults: “*Too many websites do not work well when zoomed in to make them usable by aging eyes*” [P252, 30/7 Mbps]. Another participant elaborated multiple sources of disconnection for older adults (lack of access, devices, money, know how, and help to setup), explaining how the “*barriers are endless for them*” [P159, 310/93 Mbps].

Rural participants regularly experienced frustrations related to the disconnection between unstable and unreliable Internet and cell service and the high cost they paid for this service. An older adult participant detailed the disconnect between expectations for constant upgrades and affordability,

“*I am retired. I shouldn’t be expected to keep my OS updated and pay monthly subscription fees for Adobe, MS Office, etc. in order to interact with Interior Health, City Services, the Provincial Government, yet this is exactly what is happening. Hardware and software are expensive. It’s just another barrier to entry to daily community support*” [P93, 61/5 Mbps].

Similarly, a participant with 96/1.1 speed test relayed, “*Speed is intermittent. That’s what we pay for, but upload speed rarely meets that*” (P8).

Despite this, participants had found ways to navigate the many interdependent disconnects. Some opted to remain disconnected due to cost or from lack of perceived benefit. Others advocated for connectivity to be a matter of individual choice and not mandatory, as one participant explained: “*I do believe that people should have an option to ‘opt out’ of technology if needed (i.e., virtual health care visits don’t work for some people)*” [P238, 505/24 Mbps]. Finally, some acknowledged the need for connectivity to engage in everyday life but had become selective in choosing (or filtered) what was most useful, as one participant explained:

“**If I could comfortably live without it, I would, but living in a small town limits my options. I used to believe tech was a* *necessary evil, but after getting better acquainted, I realized I can use what I need and discard the rest**” [P96, 182/129 Mbps].

## Discussion

This study examined the the experience of digital connectivity among rural-dwelling adults with and without home high-speed Internet in British Columbia, Canada. Despite reaching a diverse sample, including some with no home access to Internet, we found very few differences between those with and without access to download speeds of 50 Mbps. The lack of between group differences suggests the universal need to access the Internet with similar experiences observed in open-text responses that highlighted the many benefits alongside challenges to connectivity.

Hypotheses 1 and 2 were partially supported, as participants without access to 50 Mbps tended to be older and reside in more remote and Northern and Island regions compared to those with access, but with no differences in education or other demographic variables. Northern BC is the largest region of the province, with more dispersed rural communities whereas Island communities require subsea cable for wired Internet making these regions more costly to connect compared to the other provincial regions; however, connectivity funding is considered an investment (i.e., expenditures expected to have longer-term economic benefits) and economic benefits have been estimated to be 7 times the initial provincial investment in Northern and Island regions [31,32]. Demographic factors are often superimposed on connectivity limitations to disadvantage those living in rural communities but in the current sample participants were primarily young to middle adult age, well educated, living with family which have been linked to digital engagement [18].

The lack of significant differences between rural residents with and without home high-speed broadband in readiness, confidence, and competence (hypothesis 3) signals that other factors beyond the basic connectivity infrastructure also play an important role in rural citizens’ comfort in the digital space. Not only did predisposition and perceived ability and confidence in their skills using new technology not differ between the two groups but also perceived competence in using technology.

Hypothesis 4 was partially supported showing a paradoxical pattern in daily practices. Those below 50 Mbps were significantly more frustrated with technology and had fewer connected devices. The difference in the number of connected devices (>4 vs 3) is consistent with the differing Internet speeds, as speeds above 50/10 are required to support more than 4 devices [33]. Even though the under 50 Mbps group reported their connectivity limited their online activity more than their over 50 Mbps counterparts they used the Internet just as frequently for different activities. It’s reasonable that lower speeds meant participants’ online activities may have taken them longer to accomplish, despite the overall similar frequency of going online for each. It is also possible that using smartphones with mobile LTE [8] for online activity accounts for the lack of difference, although only 2/3 of participants reported having mobile data and often experienced challenges with accessing mobile connectivity. The one exception was the frequency of video streaming being lower among those without access to 50 Mbps. Similarly, in a rural USA community study, compared to underserved, the residents recently connected to higher speeds were able to use more devices simultaneously, and the top intended use was video streaming [34]. Indeed, speeds of 1/1 Mbps are sufficient for basic email (without large attachments), yet speeds of 30/2 Mbps are needed for streaming 4K video [35]. Overall, the lack of differences between groups speaks to the universal necessity of Internet to everyday life.

Finally, hypothesis 5 was partially supported with no difference in trust but lower ratings of Internet quality among those without home access to 50 Mbps compared to those with access. The low trust scores across both groups reflects the global trend that trust of the Internet has dropped since 2019 [36]. Positive views of connectivity expressed by both groups may over-ride concerns about security and privacy, yet trust as a reason for non-use has shifted in recent decades, with many experts suggesting lack of trust will not reduce reliance on the Internet [37]. Although connectivity quality was significantly lower among participants with under 50 Mbps, open-ended responses reflected pervasive disconnects among both groups. Digital infrastructures in rural locales are commonly unreliable or of variable quality [38,39], possibly partially obscuring the over/under 50 Mbps distinction. Indeed, we observed similarities in the open-ended responses, and found there was not a clear distinction between those above and below 50 Mbps.

This study is unique in being among the first to test novel research hypotheses guided by Ge at al.’s comprehensive approach to digital poverty [18]. This is one of the few studies exploring digital equity in the context of rural residents and using high-speed Internet access as the differentiator. Yet, counter to hypotheses, there were few differences between those with and without 50 Mbps broadband speeds. We interpret this within the context of the necessity of Internet to suggest the findings reflect that rural citizens constitute ways to engage with the digital world regardless of their digital speeds. In a recent analysis of CRTC public consultation submissions, northern Canadian residents expressed expectations for reliable, affordable and accessible connectivity [40] amid frequently reported challenges of high cost, slow, and intermittent speeds [41] Our findings comparing those with and without access to 50 Mbps suggest that this is the case among both groups, despite more pronounced connectivity limitations among those with under 50. Definitions of what constitutes “rural” appearing in the literature have been varied and sometimes conflicting, plaguing research and policy for decades [42]. Because high-speed connectivity has become so indispensable to everyday life, and rural citizens are disproportionately impacted, we suggest that indices of remoteness could begin to consider available connectivity alongside more common metrics such as population size, density, and distance to larger centers [42,43].

Our participants perceived growing expectations from all sectors (government, businesses, healthcare) for citizens to be well connected and yet for many in both groups this was not a reality. Some have begun to suggest that the 50/10 benchmark is insufficient [44]. In March 2024, the United States Federal Commission of Communications (FCC) re-defined high-speed as 100/20 Mbps, formerly 25/3 Mbps since 2015 [45]. Whether the Canadian government will similarly increase speed targets is yet to be determined, but the challenges rural participants experienced with connectivity quality, even among those with access to 50/10 Mbps, suggest that policymakers should consider reevaluating the 50/10 Mbps benchmark as a sole indicator of digital inclusion.

Further, policy should account for rural contextual factors alongside “objective” speed. Our findings align with Ge et al.’s (2022) call to look beyond the numbers when considering digital poverty and instead focus on the everyday situated practices of rural citizens and the strategies they adopt to meaningfully participate online in ways they value. A comparative analysis of information-communication technology access in rural communities in Africa and the United States reported that factors influencing Internet use differed, with economic benefit playing a central role in Africa, but entertainment and social value as larger drivers in the United States; the authors pointed to the importance of innovative, context-relevant approaches to address the multi-faceted nature of the digital divide [46]. New approaches that include community-engaged planning, monitoring, and evaluation are needed to inform effective, place-based telecommunications programming [40]. By profiling rural community residents both with and without access to high-speed broadband, we hope to advance understanding of both of these groups and their experiences in support of such approaches.

### Limitations and strengths

Diverse recruitment strategies made it difficult to determine response rates and non-response bias. The relative homogeneity of the current sample in gender and cultural identity, mirrors global and American trends but limits diversity. Income data were not collected which was a limitation as in previous work low income has been related to digital poverty [18,47]. Although we had some representation across gender and education groups, the predominance of well-educated women participants may have shaped the results (i.e., that we found few differences between those with and without high-speed internet). More research is needed with people facing socioeconomic disadvantage, who may face the greatest challenges related to access to highspeed Internet and impacts on digital poverty. Future recruitment through paid means, such as at local centres or targeted strategies to promote gender and cultural diversity, with various levels of access, income, and education are recommended.

The majority of our participants completed online surveys, which may introduce potential bias by excluding residents with inadequate Internet access or broadband connectivity, limiting generalizability of the findings. Nevertheless, we reached participants from very remote locations, suggesting an approximation of a representative sample with respect to rurality. Our online survey was accessible to those with low Internet speeds, and in Canada, only a small proportion of rural and remote Canadians have no access to Internet services (unserved) [9]. Importantly, research has highlighted the underrepresentation of the voices of small and very remote communities [41], which we mitigated through the use of the National Broadband Internet Service Availability Map and Canada Post’s neighborhood postal code targetor tool to ensure remote locations with low broadband coverage were reached with both print surveys and targeted community advertisements. However, it’s possible that we over-represented participants with low and inadequate broadband speeds. Our decision to group based on download speeds may have limited optimal group distinctiveness reflected in some individuals who reported access to 50 Mbps with low quality and reliability.

Even considering these limitations, this study captured a comprehensive cross-section of rural participants from diverse rural geographical locations in the province. This inclusivity provided multiple and diverse perspectives that might not have been uncovered in a narrower sample of participants.

## Conclusion

Despite similar digital readiness, mobile device proficiency, self-efficacy, trust, and frequency of Internet use, participants with below 50 Mbps download speeds at home had higher frustration with Internet-connected technology, reported having fewer connected devices, rated their Internet quality lower, and reported being more limited in online activity compared to those with access to 50 + Mbps. Yet, rural participants both with and without access to 50 Mbps alike cited the actual and potential benefits of high-speed connectivity, and experienced disconnects on many levels, but particularly between expectations for and reality of high-speed connectivity.

## Supporting information

S1 File STROBE-checklist-Rural-Broadband-PLOS.doc. STROBE Statement—Checklist of items that should be included in reports of cross-sectional studies.
(DOC)
